# Osteoradionecrosis prevalence and associated factors: A ten years retrospective study

**DOI:** 10.4317/medoral.22310

**Published:** 2018-11-21

**Authors:** Igor-Figueiredo Pereira, Ramon-Targino Firmino, Henrique-Côrtes Meira, Belmiro-Cavalcanti-Do Egito Vasconcelos, Vladimir-Reimar-Augusto-de Souza Noronha, Vagner-Rodrigues Santos

**Affiliations:** 1 Doctor Student - School of Dentistry, University of Pernambuco. Department of Prosthodontics and Bucco-Facial Surgery. Newton Cavalcanti, 1650 - 54753-020 – Inabi, Camaragibe – PE, Brazil; 2 Doctor Student - School of Dentistry, Federal University of Minas Gerais. Department of Pediatric Dentistry and Orthodontics. Presidente Antônio Carlos, 6627 - 31270-901- Pampulha Belo Horizonte - MG, Brazil; 3 Master - School of Dentistry, Federal University of Minas Gerais. Department of Clinical, Pathology and Surgery. Presidente Antônio Carlos, 6627 - 31270-901- Pampulha Belo Horizonte - MG, Brazil; 4 Doctor - University of Pernambuco. Department of Prosthodontics and Bucco-Facial Surgery, Newton Cavalcanti, 1650 - 54753-020 – Inabi, Camaragibe – PE, Brazil; 5 Doctor - University Center Newton Paiva. Department of surgery. R. Mal. Foch, 15 - 30431-189, Grajaú, - Belo Horizonte – MG, Brazil; 6 Doctor - School of Dentistry, Federal University of Minas Gerais. Department of Clinical, Pathology and Surgery. Presidente Antônio Carlos, 6627 - 31270-901- Pampulha Belo Horizonte - MG, Brazil

## Abstract

**Background:**

Osteoradionecrosis (ORN) is one of the most serious complications of head and neck radiotherapy and is considered a public health problem worldwide. This study aims to determine the prevalence and associated factors of ORN in patients undergoing radiotherapy for head and neck malignancy.

**Material and Methods:**

A cross-sectional retrospective study was conducted, in which all medical records of patients undergoing head and neck radiation in the period between 2006 to 2015 (10 years) were examined. Clinical and demographic data were extracted. Multivariate Poisson regression analysis with robust variance was employed to access the relationship between ORN and independent variables (*p*<0.05; 95% CI).

**Results:**

The sample comprised 413 medical records of patients undergoing radiotherapy. The prevalence of ORN was 9.7 %. Most participants were males (78.2%). The mean age of subjects was 55 years (± 14 years). The mandible was the main site of occurrence of ORN (85.0%). The following variables were associated with ORN : presence of oral mucositis (PR = 3.03; 95% CI: 1.30-7.03), history of smoking (PR = 0.23; 95% CI: 0.07-0.74), number of teeth removed before radiotherapy (PR = 1.06; 95% CI: 1.01-1.11) and visit to the dentist before radiation (PR = 0.08; 95% CI: 1.02-1.11).

**Conclusions:**

The prevalence of ORN was low and was associated with the presence of oral mucositis and the number of removed teeth before radiation. Visiting the dentist before radiotherapy and stop-ping smoking were protective factors for ORN.

** Key words:**Head and neck neoplasms, osteoradionecrosis, radiotherapy, adjuvant chemo-therapy, epidemiology.

## Introduction

Osteoradionecrosis (ORN) is one of the most serious complications of head and neck radiotherapy and is considered a public health problem worldwide (1). It is characterized by defects in healing and the loss of bone viability, induced by the tissue effects of radiation (2).

The mandible is the most affected bone, as it is frequently present in the radiation field, which exerts an important influence on the development of ORN (3). Initially, ORN may appear as asymptomatic bone changes, with decreased bone density in the irradiated region, delayed healing and destruction of the cortical bone. However, it is most commonly characterized by the exposure of the affected bone, in addition to oral ulcers, drainage of purulent secretion and oral fistulas. Pain, foul odor, discomfort in masticatory, swallowing, and speech difficulties are the most common complications. In some cases, pathological fractures have also be observed (4).

The prevalence and incidence of ORN have been reported in the literature with a variation of 5-10% (5,6). In addition, several risk factors have been suggested for the onset of ORN, with emphasis on the post-radiotherapy exodontia. Important studies indicate the importance of integrated treatment plan for these patients (7). The objective of this study was to analyze the prevalence of ORN in the jaws, in a 10-year retrospective study, as well as the associated factors.

## Material and Methods

A cross-sectional, retrospective, and analytical study was carried out at the Dentistry School of the Federal University of Minas Gerais, Belo Horizonte, Brazil, a reference center in the dental care of patients with head and neck cancer. The study sample comprised the medical records of all patients with head and neck cancer treated between 2006 and 2015, who underwent radiotherapy.

-Data collection

The records were carefully examined by a previously trained researcher, who searched the following variables: age, gender, skin color, type of malignancy, location of lesion, alcohol and tobacco consumption history, radiotherapy (total dose, number of sessions), bone affected and number of dental extractions performed prior to radiotherapy.

-Data analysis

Data were analyzed using descriptive and inferential statistics, applying the Statistical Package for Social Sciences (SPSS for Windows, version 23.0, IBM Inc., Amonk, NY, USA). After having performed a descriptive analysis, a Poisson multiple regression analysis with robust variance was performed, in which an association between the dependent variable (osteoradionecrosis) and the independent variables was adopted, respecting a 95% confidence level (*p*<0.05). The variables with *p*<0.20 in the bivariate analysis were included in the multiple analysis; those with *p*<0.05 were maintained in the final model.

-Ethical aspects

The present study was carried out following the ethical precepts of Resolution 466/12 of the National Health Council, as well as those of the Declaration of Helsinki. Information was only accepted from medical records that had been correctly filled out, that contained the patients’ authorizations, and that had a free and informed consent form that had been properly signed. This study was approved by the local Research Ethics Committee (protocol: CAAE-47197715.0.0000.5149).

## Results

The study evaluated 413 charts of patients submitted to head and neck radiotherapy between January 2006 and December 2015.  Table 1 presents the characteristics of the study. The prevalence of ORN was 9.7% (n = 40 cases). The majority of the patients were male (78.2%) and had a mean age of 55 years (± 14). Squamous cell carcinoma (76.3%) was the most common type of cancer, predominantly affecting the oral cavity (42.2%). On average, patients received a radiation dose of 60.43 Gy (± 8.23) and removed 9.9 teeth (± 6.5). ORN proved to be more frequent in the mandible (85%).

**Table 1:**
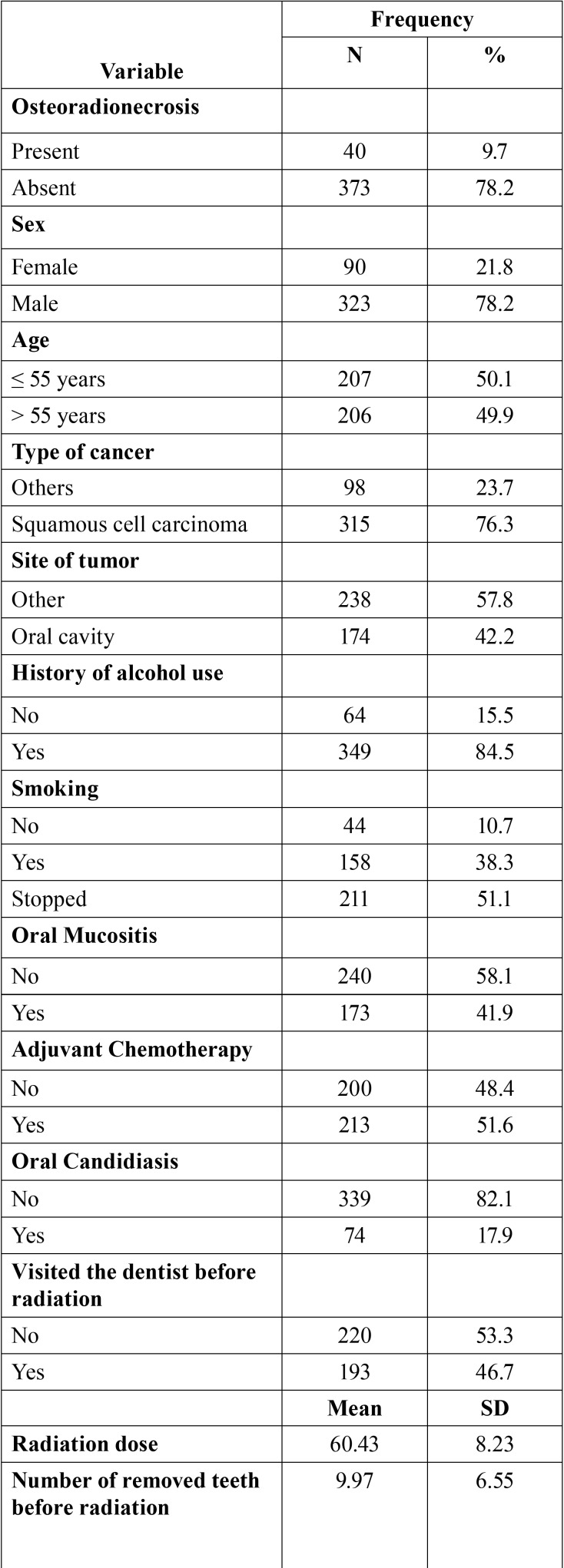
Characteristics of the 413 patients submitted to head and neck radiotherapy in a ten years period.

 Table 2,  Table 2 continue shows the ORN frequency according to the independent variables. In the final model, ORN presence was associated with the presence of oral mucositis (OM) (PR = 3.03, 95% CI: 1.30-7.03), history of smoking (PR = 0.23, 95% CI: 0.07-0.74), number of teeth removed (PR = 1.06; 95% CI: 1.01-1.11) and visits to the dentist before radiotherapy (PR = 0.08; 95% CI: 1.02-1.11).

**Table 2:**
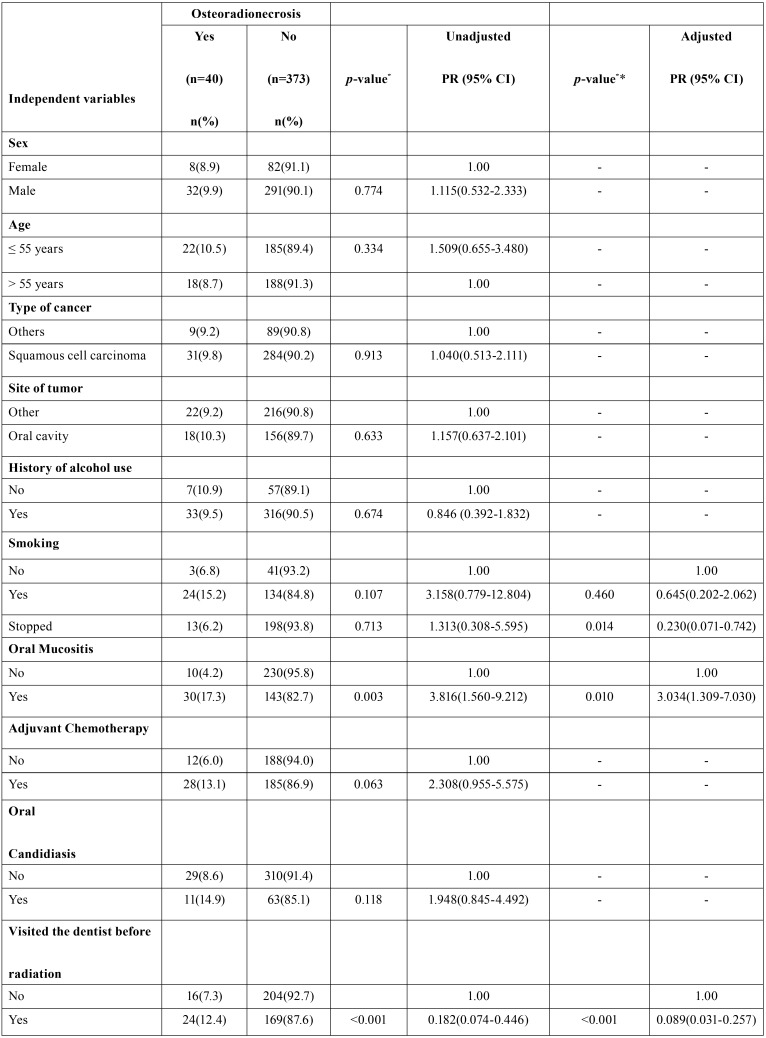
Bivariate and multivariate Poisson regression analysis regarding the association of ORN and independent variables among patients undergoing radiotherapy.

**Table 2 continue:**
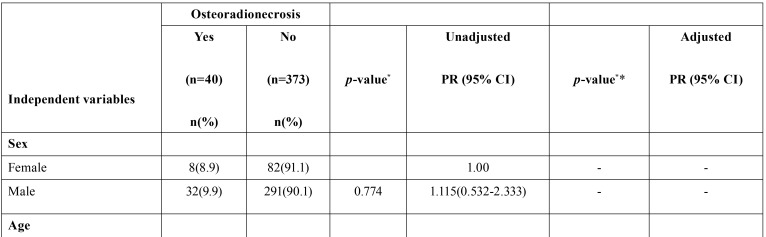
Bivariate and multivariate Poisson regression analysis regarding the association of ORN and independent variables among patients undergoing radiotherapy.

## Discussion

Osteoradionecrosis of the jaws is one of the major complications of radiotherapy in the head and neck region. Despite of protective actions, it can not always be avoided, causing psychological and physical impact on cancer patients (6).

A total of 413 medical records of patients with malignant head and neck neoplasms were evaluated over a 10-year period, with 40 cases of osteoradionecrosis (9.7%) diagnosed. There is a great variation in the prevalence and incidence of ORN in several studies, but the result observed in the present study is similar to more recent researches, which predominately identify the frequency of ORN between 4-10% (5,8). In addition, the risk of developing ORN has declined in recent years due to advances in radiotherapy and the introduction of pre-radiotherapy dental care (9). However, it is difficult to compare the present data with other studies due to a lack of standardization of diagnoses and classifications of ORN, different tumor locations, varied radiation/dosing techniques and sample size.

Although not statistically different, individuals diagnosed with ORN were mostly men, with a mean age of 55 years. Other studies also found similar demographic data, where 72.7% - 76.0% were men, categorized in the fifth decade of life (6,10,11). This higher occurrence in the male sex can be justified by the harmful habits practiced by men, such as higher consumption of alcohol and tobacco and less care with oral hygiene (10,12).

The main site of primary tumors in the head and neck region was the oral cavity (45%), with squamous cell carcinoma being the most frequent (77.5%). Several authors also found the main localization of the tumor in the oral cavity, ranging between 53.6% (10), 60.6%(6) and 74%(11). Tumor location is an important factor in ORN risk prediction, because the radiotherapy of tumors in the oral cavity or oropharynx probably includes the mandible in the radiation field (9). 

The mandible was most affected by ORN than maxilla (85%). According to the previous studies, the mandible is affected in approximately 90% of the cases (6,13). It has a greater risk of developing osteoradionecrosis than the maxilla because of the difference in anatomy and bone density, as well as the lower blood supply (6,9,14).

The pathogenesis and etiology of ORN have been explained by several hypotheses. The most accepted is the theory of “hypoxia, hypocellularization, and hypovascularization” (13). In addition, several risk factors have been reported to the development of ORN: radiation dose, pre-radiotherapy surgery, associated chemotherapy, oral health status, dental surgery, tumor location, comorbidities and alcohol and tobacco abuse (5,9-11). In the present study, the following variables presented a statistically significant association with ORN: the presence of oral mucositis, history of smoking, dental extraction before to radiotherapy and having gone to the dentist before radiotherapy.

A previous study found a prevalence of oral mucositis in irradiated patients with head and neck neoplasms is approximately 80% (15), much higher than the present study (41.9%). In this study, patients with oral mucositis had a prevalence of ORN approximately three times greater. Few studies shows the relation between the two complications, since oral mucositis is an acute condition, whereas ORN is a chronic condition. However, this relationship can be explained since both conditions have a common risk factor: elevated dose of radiation and the association with chemotherapy and radiation field (16).

The oral health status of patients with head and neck neoplasms is much more unfavorable than the general population, due to alcohol and tobacco abuse, coupled with poor oral hygiene (5,17). Patients with poor oral hygiene and dental extractions after radiotherapy are associated with an increased risk for the development of ORN (11).

An average of 9.97 teeth were removed before radiation. The present study found that exodontia performed before radiotherapy is a factor associated with ORN. This result must be interpreted with caution, as despite statistically significant, the low difference between groups suggests that this variable may be not clinically important. Although radiotherapy should begin at least 15 days after oral surgery, this interval is not always obeyed due to the severity of the cancer. However, visiting the dentist before radiation therapy was a protective factor.

The excessive consumption of alcohol and tobacco is another risk factor for ORN. A previous study observed a 32% increase in the incidence of ORN in patients who smoked during radiotherapy (11,18,19). In the present study, 82.5% of the patients with ORN had a history of alcohol use and 92.5% had a history of tobacco use. It was also observed that stopping smoking was a protective factor for ORN. Patients who stopped smoking presented significantly less ORN, a fact that can be explained by tobacco interference in healing processes, as well as aggression to the oral mucosa (8).

Despite being limited by the cross-sectional design, the results of this investigation should not be disregarded. Our findings reinforce that the use of oral adequacy protocols prior to radiochemotherapy treatment, is essential to prevent complications such as ORN. Removing compromised teeth before radiotherapy is as important as respecting a minimal period of alveolar healing.
